# Case Report of Suspected Gonadal Mosaicism in *FOXP1*-Related Neurodevelopmental Disorder

**DOI:** 10.3390/ijms25115709

**Published:** 2024-05-24

**Authors:** Anna Zsigmond, Ágnes Till, Judit Bene, Márta Czakó, Alexandra Mikó, Kinga Hadzsiev

**Affiliations:** 1 Department of Medical Genetics, Medical School, University of Pécs, H-7623 Pécs, Hungary; zsigmond.anna@pte.hu (A.Z.); till.agnes@pte.hu (Á.T.); bene.judit@pte.hu (J.B.); czako.marta@pte.hu (M.C.); miko.alexandra@pte.hu (A.M.); 2 Institute for Translational Medicine, Medical School, University of Pécs, H-7624 Pécs, Hungary

**Keywords:** *FOXP1* gene, neurodevelopmental disorder, gonadal mosaicism, speech delay

## Abstract

Heterozygous mutations in the *FOXP1* gene (OMIM#605515) are responsible for a well-characterized neurodevelopmental syndrome known as “intellectual developmental disorder with language impairment with or without autistic features” (OMIM#613670) or FOXP1 syndrome for short. The main features of the condition are global developmental delay/intellectual disability; speech impairment in all individuals, regardless of their level of cognitive abilities; behavioral abnormalities; congenital anomalies, including subtle dysmorphic features; and strabismus, brain, cardiac, and urogenital abnormalities. Here, we present two siblings with a de novo heterozygous *FOXP1* variant, namely, a four-year-old boy and 14-month-old girl. Both children have significantly delayed early psychomotor development, hypotonia, and very similar, slightly dysmorphic facial features. A lack of expressive speech was the leading symptom in the case of the four-year-old boy. We performed whole-exome sequencing on the male patient, which identified a pathogenic heterozygous c.1541G>A (p.Arg514His) *FOXP1* mutation. His sister’s targeted mutation analysis also showed the same heterozygous *FOXP1* variant. Segregation analysis revealed the de novo origin of the mutation, suggesting the presence of parental gonadal mosaicism. To the best of our knowledge, this is the first report of gonadal mosaicism in *FOXP1*-related neurodevelopmental disorders in the medical literature.

## 1. Introduction

Neurodevelopmental disorders (NDDs) represent a serious health problem, affecting >3% of children worldwide. These disorders are characterized by an inability to reach cognitive, emotional, and motor developmental milestones. NDDs encompass intellectual disability, autism spectrum disorder, attention deficit hyperactivity disorder, and epilepsy, among other issues. They are the result of the interruption of essential neurodevelopmental processes. There are many genes and mutations that are associated with NDDs, confirming their heterogeneous origins [1]. The widespread use of array comparative genomic hybridization (CGH) and next-generation exome- and genome-sequencing approaches has provided new perspectives in the genetic diagnosis of NDDs. However, many individuals with NDDs still do not obtain molecular diagnoses. The early diagnosis of patients with NDD is mandatory for genetic counselling, multidisciplinary management, and medical intervention. The *FOXP1*-related neurodevelopmental disorder is one of the most recently recognized NDDs.

The forkhead box protein 1 (FOXP1) was first characterized in 2001 as a member of a winged helix/forkhead transcriptional factor family [2]. FOXP1 plays an important part in early embryogenesis, and its function is fundamental in cell metabolism [3]. The *FOXP1* gene is located on chromosome 3p13 and its product, the FOXP1 protein, is a transcriptional factor which plays an important role from the early phase of development. The FOXP1 protein is widely expressed. It is required for the development of the brain, spinal motor neurons, lung, and esophagus [4,5,6,7,8]. It also plays a role in the cardiac growth and regulation of macrophage differentiation [9]. 

The *FOXP1*-related neurodevelopmental disorder was described as a recognizable entity with a wide clinical spectrum [10]. Mutations or deletions that disrupt the *FOXP1* gene cause the autosomal dominantly inherited “intellectual developmental disorder with language impairment with or without autistic features” disease (OMIM#613670) or *FOXP1*-related neurodevelopmental disorder (or FOXP1 syndrome for short). The main features of the condition are global developmental delay/intellectual disability, speech impairment, behavior abnormalities, and congenital anomalies. Common congenital abnormalities associated with the disorder include subtle dysmorphic features, strabismus, excessive drooling, and brain, cardiac, and urogenital abnormalities. To date, more than 200 individuals have been identified as having FOXP1 syndrome [11]. A systematic review conducted in 2021 summarized these cases and suggested practical parameters for the clinical assessment and monitoring of the affected patients [12]. Thus far, around 101 *FOXP1*-related variants have been reported in patients based on the HGMD database, including 38 gross deletions/insertions, 33 missense/nonsense variants, 17 small deletions/insertions, 1 small indel, 9 splicing variants, and 3 complex rearrangements [13]. Most patients with FOXP1 syndrome, when their parents undergo molecular genetic testing, have the disorder as the result of a de novo pathogenic variant. 

However, studies generally suggest a 1–2% overall risk for gonadal mosaicism for point mutations and a risk of up to 4% for chromosomal rearrangements [14]. *FOXP1*-related NDD is inherited in an autosomal dominant mode, and gonadal mosaicism has not been reported previously. Here, we present the case of a family with two affected children from our Rare Disease Center, where the possibility of parental gonadal mosaicism of a pathogenic *FOXP1* gene mutation was suggested. 

## 2. Case Presentation

### 2.1. Patient 1

**Figure 1:**
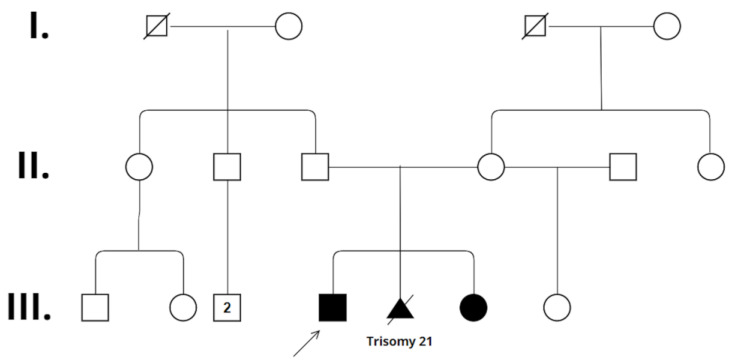
Pedigree of the family. Standardized human pedigree nomenclature is used. A square represents a male, and a circle represents a female. The relationship line is a horizontal line between two people. A vertical line is drawn from parents to children and is called a line of descent. The sibship line is a horizontal line joining brothers and sisters. Roman numerals symbolize generations, beginning with the oldest. The symbols of the affected children are colored. The arrow represents the elder affected child. The termination of pregnancy is marked by a triangle with a line drawn through the symbol. This symbol is also colored since the fetus was affected with a genetic condition.

**Figure 2:**
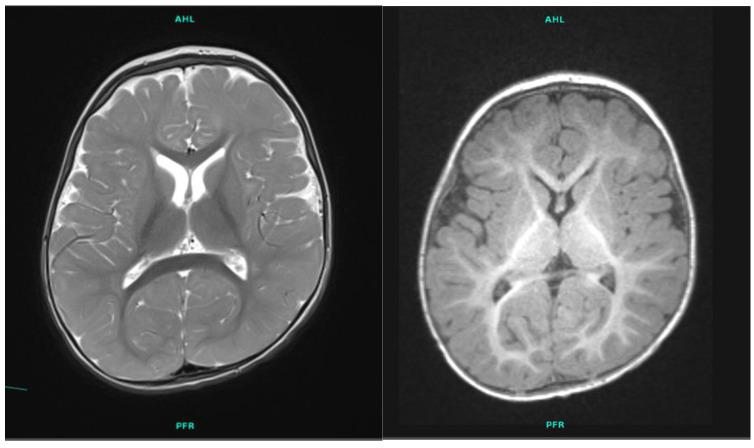
T2 and T1 weighted axial brain MRI images of patient 1 showing smaller basal ganglia and cerebral atrophy with fronto-temporal dominance.

The four-year-old boy is the first child of the 41-year-old father and the second child of the 31-year-old mother (Figure 1). The parents are healthy and non-consanguineous. The family history is negative for developmental delay, speech delay, intellectual disability, or any other neurological or psychiatric disease. The boy’s elder maternal half-sister is also healthy. The patient was born during the 41st week of gestation by cesarean section with a birth weight of 3600 g (50–75 percentile). His postnatal adaptation was uneventful. His developmental milestones were delayed. He turned over at 10 months, crawled at 10 months, and walked alone at 2.5 years. His expressive language development was delayed as well. His cognitive developmental quotient when evaluated at 28 months of age was 60, his language developmental quotient was 56, and his motor developmental quotient was 55, as determined using the Bayley Scales of Infant and Toddler Development Test, third edition (BSID III). Laboratory tests and metabolic evaluation (including urine organic acids, serum amino acids, and thyroid function tests) were normal. The electroencephalography (EEG) result was negative. Magnetic resonance imaging (MRI) scanning of the brain showed smaller basal ganglia and cerebral atrophy with fronto-temporal dominance (Figure 2). At 18 months of age he was referred to genetic counselling due to global developmental delay. Somatic parameters were age-appropriate, the weight was in the 10–25 percentile, the height was in the 50–75 percentile, and the head circumference was 10–25 percentile. Neurological examination revealed mild axial hypotonia, convergent strabismus, and some dyskinetic movements. His facial dysmorphic features were subtle: a prominent forehead, a frontal hair upsweep, a wide nasal bridge with a broad nasal tip, and ocular hypertelorism were observed. The patient was unable to walk alone and was only able to babble. 

Moderate intellectual disability with autistic features and a lack of expressive speech were later observed in this patient. His cognitive developmental quotient, evaluated at 42 months of age, was 65, his language developmental quotient was 54, and his motor developmental quotient was 58, as determined using BSID III. Psychiatric examination is currently in progress for autism spectrum disorder. 

### 2.2. Patient 2

**Figure 3:**
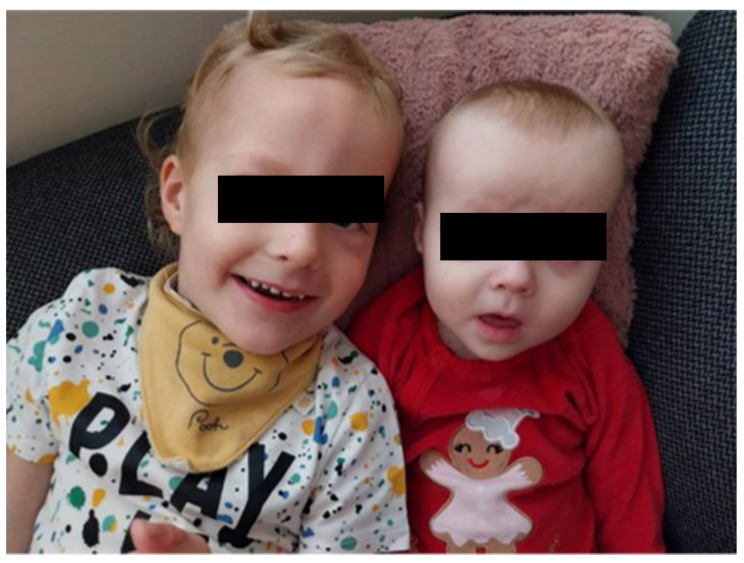
Facial characteristics of the siblings. Both children have prominent foreheads, frontal hair upsweep, ocular hypertelorism, wide nasal bridge with broad nasal tip, and convergent strabismus.

The younger sister of the patient presented above is the second common child of the healthy, non-consanguineous couple; she is the third child of the mother and the second child of the father. She was born after a previous pregnancy termination following the prenatal diagnosis of 21 trisomy. She was born by cesarean section at 39th week of gestation with a birth weight of 3630 g (95 percentile) after an uneventful pregnancy. Her postnatal adaptation was uneventful. Moderate generalized hypotonia was observed at birth, and global developmental delay became evident later. She was referred for genetic counselling at 8 months of age. She had feeding difficulties, her weight gain was below the level for her age (3–10 percentile), and her height and head circumference were in the normal range (both 25–50 percentile). Neurological examination revealed generalized hypotonia and bilateral convergent strabismus. She had similar facial characteristics as her brother (wide forehead, frontal hair upsweep, wide nasal bridge, round face, and ocular hypertelorism) (Figure 3). She could not crawl or sit alone; she did not reach for toys and visual attention was also poor. Babbling was also absent. 

Her cognitive developmental quotient when evaluated at 14 months of age was 75, her language developmental quotient was 74, and her motor developmental quotient was 61, as determined using BSID III, thus proving global developmental delay. 

## 3. Results

Chromosome-based analysis of the brother showed a normal male karyotype. Angelman syndrome fluorescent in situ hybridization (FISH) and array comparative genomic hybridization (array CGH) also showed normal results. Whole-exome sequencing (WES) identified a pathogenic heterozygous c.1541G>A (p.Arg514His) FOXP1 mutation [NM_032682.6]. This variant results in an arginine-to-histidine substitution within the DNA recognition helix of the FOX DNA-binding domain. The severity of the mutation was assessed using PolyPhen-2 and found to probably be damaging, involving the disruption of multiple protein functions. This variant leads to aberrant subcellular localization and the loss of transcriptional repression activity, exerting the effect of protein interactions [15]. 

Targeted mutation analysis of the younger sister showed the same heterozygous FOXP1 variant as her brother’s. 

The segregation analysis proved the de novo origin of the mutation, suggesting parental mosaicism. The father did not agree to conduct sperm analysis; thus, we could not prove the paternal or maternal origin of the gonadal mosaicism. 

## 4. Discussion

Here, we present two siblings with a de novo heterozygous FOXP1 variant. Both children had delayed psychomotor development, generalized hypotonia, and subtly different but very similar facial features (Table 1). A lack of expressive speech is the leading symptom of the brother. 

WES analysis of the brother identified a pathogenic heterozygous c.1541G>A (p.Arg514His) FOXP1 mutation [NM_032682.6]. His sister’s targeted mutation analysis also showed the same heterozygous missense FOXP1 variant. The siblings had phenotypes that were consistent with the previously reported cases of FOXP1 syndrome. Segregation analysis using DNA isolated from the parent’s peripheral blood samples revealed the de novo origin of the mutation. The fact that the children with the same heterozygous FOXP1 variant were born to healthy, non-carrier parents suggests the presence of parental gonadal mosaicism. During genetic counselling, parents were informed about the limitation of the segregation analysis performed from peripheral-blood-derived parental DNA samples, and we offered preimplantation genetic testing or prenatal genetic testing for future pregnancies. 

It has been long known that de novo single-nucleotide variants arise in paternal germ cells three to four times more often than they do in maternal germ cells. Children of older fathers are therefore at greater risk of developing de novo mutations [16]. De novo mutations cause developmental disorders, with an estimated average birth prevalence from 1 in 213 to 1 in 448. This equates to almost 400,000 children being born every year with the condition [17]. In the cases presented here, the father was 41 years old at the birth of the older child and 45 years old at the birth of the younger child. De novo mutation can arise in the parental germline during embryonic and fetal development or postnatally. The timing of the mutation will define its clinical consequences [18]. In practice, it is difficult to distinguish between parental gonadal mosaicism, de novo gonadal issues, or early post-zygotic mutation [19], although the recurrence risk is different in these cases. De novo variants are associated with low, about 1%, recurrence risk. During genetic counselling, the clinical geneticist relies on empirical data regarding the recurrence risk for future pregnancies. The real prevalence of gonadal mosaicism is usually unknown in rare autosomal dominantly inherited NDDs. However, the rate of gonadal mosaicism may modify the recurrence risk: it can be lower, but it can be even higher. Segregation analysis is usually performed on the DNA isolated from the parent’s peripheral blood sample. Previous studies have tested parental mosaicism in peripheral blood and sperm samples, reporting various levels of somatic and gonadal mosaicism [20,21,22]. The detection of maternal gonadal mosaicism from oocytes causes technical difficulties, since oocytes can be obtained only by invasive procedure. However, sperm analysis is a possible method of detecting paternal gonadal mosaicism that was studied recently [20,22,23,24,25]. In a Chinese Dravet syndrome cohort, Yang et al. reported higher levels of *SCN1A* mosaicism in sperm samples compared to blood samples of mosaic fathers [25]. Frisk et al. investigated the prevalence of mosaicism in parents of children with intellectual disability syndromes caused by de novo variants and performed droplet digital PCR on DNA extracted from blood and sperm. They detected low-level *EHMT1* mosaicism in the sperm sample of a father whose blood sample did not show mosaicism and a higher level of mosaicism was detected in the sperm sample of a father who showed the *ITPR1* mosaic variant in a blood sample [20]. Last year, Wen at al. reported on the paternal *MECP2* gonadal mosaicism of nine fathers’ daughters with Rett syndrome, and in only one of case was mosaicism identified in the blood sample. The research team also found gonadal mosaicism in the sperm samples of healthy males who had no Rett syndrome relatives, and none of these individuals had mosaicism in blood-derived DNA [24]. A Hungarian case report by Bessenyei et al. reported paternal gonadal mosaicism for *MED13L*-related disorders. A healthy father with two affected children from different relationships was presented. One of the children had a missense *MED13L* mutation, which was not detected from the father’s blood-sample-derived DNA. However, the child had an elder affected sibling, whose mother did not consent to his genetic testing. The similarity between the two affected siblings raised the possibility of gonadal mosaicism. Sperm analysis identified the presence of a high rate *MED13L* mutation (30–50%) of the father’s sperm cells, confirming paternal gonadal mosaicism [26]. 

The *FOXP1*-related neurodevelopmental disorder was described as a recognizable entity with a wide clinical spectrum in 2017 [10]. Since the phenotypic features associated with FOXP1 syndrome are not sufficient to perform the diagnosis, all NDDs should be considered in the differential diagnosis. The recommended molecular genetic testing approaches include the use of array CGH, an intellectual disability multigene panel, or comprehensive genomic testing, like WES or genome sequencing. A small number of cases with FOXP1 syndrome cases are known in the current medical literature; this number may increase in the future with the widespread use of comprehensive genetic testing. Only a few studies deal with the examination of parental gonadal mosaicism. To the best of our knowledge, there are no previous reports of gonadal mosaicism in *FOXP1*-related neurodevelopmental disorder in the medical literature. In addition to a definitive molecular genetic diagnosis in rare genetic disorders, the reporting of mosaic cases is also critically important. 

## 5. Methods

### 5.1. GTG Banding

Karyotyping was carried out by Giemsa–Trypsin (GTG) banding from peripheral blood lymphocytes using standard procedures [27]. To exclude mosaicism, 100 cells were analyzed in each case. 

### 5.2. FISH

The UBE3A locus (Prader–Willi/Angelman Critical Region)-specific probe was applied for FISH examination using controls in 15p11.2 and 15q22 regions, respectively (Vysis, Abbott Laboratories, Abbott Park, IL, USA) [28]. The protocol used was in accordance with the manufacturer’s instructions.

### 5.3. Array CGH

Array CGH was performed using Agilent Human Genome Sureprint G3 8x60K oli-go-array (Amadid 021924) (Agilent, Santa Clara, CA, USA) [29]. The procedures were carried out according to the manufacturer’s protocols. DNA was isolated from peripheral blood leukocytes with an E.Z.N.A.^®^ Blood DNA Maxi kit (Omega BIO-TEK, Norcross, GA, USA) according to the manufacturer’s protocol. For measuring the concentration and purity of extracted DNA, a NanoDrop 2000 spectrophotometer (Thermo Fisher Scientific, Waltham, MA, USA) was applied. DNA sequence information refers to the public UCSC database (GRCh37/hg19).

### 5.4. WES

Whole-exome sequencing was performed using DNA samples obtained from peripheral leukocytes. Exomic libraries were prepared using the Twist Human Core Exome Kit Library Prep Kit, and sequencing was performed on an Illumina NovaSeq 6000 instrument according to the manufacturer’s protocol using paired-end 100 bp reads. The mean sequencing depth of target regions was 120X. The reads were aligned to the human reference genome (GRCh37:hg19) using the Burrows–Wheeler Aligner.

For the classification and interpretation of the genomic data, the guidelines of the American College of Medical Genetics and Genomics (ACMG) were followed [30]. Moreover, databases, such as ClinVar, The Genome Aggregation Database (gnomAD) Genomes/Exomes coverage, in silico prediction tools, such as Mutation taster, PhastCons and PhyloP were used. All of the potential variants were then manually searched in the literature using PubMed and Online Mendelian Inheritance in Man (OMIM), leading to the identification of the presented variants.

### 5.5. Targeted Mutation Analysis

Targeted mutation analysis was conducted for the proband’s sister and segregation analysis was performed using DNA samples obtained from peripheral leukocytes. The variant found in the elder sibling’s DNA sample was identified via Sanger sequencing performed on the family members’ DNA samples.

## 6. Conclusions

The siblings presented here are affected by an extremely rare genetic condition. Their detailed clinical presentation contributes important information to the understanding of the *FOXP1*-related neurodevelopmental disorder.

Our cases additionally highlight the importance of gonadal mosaicism. We could not clearly prove the presence of parental gonadal mosaicism in the family. Since it is very unlikely that two same mutations develop as a new mutation within one family, the siblings’ genotypes strongly suggest the presence of parental gonadal mosaicism.

Parental gonadal mosaicism does not affect the parent’s own health status, but it can result in cases of severe disease in consecutive offsprings. The rate of gonadal mosaicism can modify the recurrence risk, which is why more clinical attention is needed for the detection. Parents are usually offered parental testing from blood samples by their clinical geneticist, and the clinical geneticist relies on empirical data regarding the recurrence risk for future pregnancies. However, analysis of DNA extracted from peripheral blood alone may underestimate gonadal mosaicism. Oocytes can only be obtained by invasive procedures and are only examined one by one; therefore, maternal gonadal mosaicism cannot be tested accurately. In contrast, sperm analysis is a possible alternative means to detect paternal gonadal mosaicism. Since most of the de novo variants arise in the paternal haplotype, sperm analysis could be offered to fathers whose child is diagnosed with de novo variant. This would be an easy-to-use examination that is not associated with high costs. Currently, this is not offered as part of routine genetic counselling. Sperm analysis would allow the clinical geneticist to give more accurate information about the rate of parental gonadal mosaicism and of the recurrence risk for future pregnancies.

## Figures and Tables

**Table 1:** Clinical characteristics of the siblings.

	Patient 1	Patient 2
Age	4 years	14 months
Sex	male	female
*FOXP1* variant	c.1541G>A heterozygous	c.1541G>A heterozygous
Neurobehavioral features		
History of hypotonia	+	+
Motor delays	+	+
Speech delays	+	+
Swallowing or feeding problems	-	+
ID or GDD	+	+
ASD features	+	-
Behavioral problems	+	-
Dysmorphic features		
Head and facial features	prominent forehead frontal hair upsweep	prominent forehead frontal hair upsweep round face
Eyes	ocular hypertelorism	ocular hypertelorism
Ears	-	-
Nose	wide nasal bridge broad nasal tip	wide nasal bridge broad nasal tip
Mouth	-	-
Extremities	-	-
Other	-	-
Medical features associated with FOXP1 syndrome		
Neurology	dyskinetic movements	-
EEG	negative	N/A
Brain MRI	smaller basal ganglia and cerebral atrophy	N/A
Endocrinology	N/A	N/A
Cardiac	N/A	N/A
Nephrology/urology	-	N/A
Ophthalmology	convergent strabismus	convergent strabismus
Other medical problems	-	-

*ID*—intellectual disability; *GDD*—global developmental delay; *ASD*—autism spectrum disorder.

## Data Availability

The data presented in this study are available on request from the corresponding author.
